# The mediating effect of family resilience between coping styles and caregiver burden in maintenance hemodialysis patients: a cross-sectional study

**DOI:** 10.1186/s12882-024-03520-2

**Published:** 2024-03-05

**Authors:** Qianjun Zhang, Qiaoling Liu, Li Zhang, Yabin Jin, Xia Xiang, Xuefang Huang, Jiezhen Mai, Tingfen Zhao, Wen Cui

**Affiliations:** 1 School of Nursing, College of Medicine, Shantou University, Shantou, China; 2 Department of Nursing, Shenzhen Hospital, Beijing University of Chinese Medicine, Shenzhen, China; 3 Department of Office, First People’s Hospital of Foshan, Foshan, China; 4 School of Computer and Communication Engineering, University of Science and Technology Beijing, Beijing, China; 5 Department of Nursing, First People’s Hospital of Foshan, Foshan, China; 6 Hemodialysis Center, First People’s Hospital of Foshan, Foshan, China; 7 Department of Hepatopancreatic Surgery, First People’s Hospital of Foshan, Foshan, China; 8 College of Nursing, Zunyi Medical University, Zhuhai, China

**Keywords:** Maintenance hemodialysis, Caregiver burden, Coping style, Family resilience

## Abstract

**Background:**

Primary caregivers of hemodialysis patients suffer from varying degrees of stress from their patients. Caring for hemodialysis patients can expose caregivers to many problems, leading to an increased burden of care and even impacting the quality of care. The purpose of our study was to examine whether family resilience could be a mediating variable moderating the relationship between patient coping styles and caregiver burden.

**Methods:**

The study was a cross-sectional and descriptive-analytical study that interviewed 173 pairs of hemodialysis patients and their caregivers at a blood purification center in a public hospital in China. The Brief Coping Styles Scale (Chinese version) was used to assess individuals’ coping styles for disease and treatment. From the caregiver’s perspective, the Family Resilience Assessment Scale (Chinese version) was used to understand the resilience of families, and the Zarit Caregiver Burden Scale was used to capture the caregiver’s subjective experience of burden. Statistical analyses were conducted using SPSS version 23 and Amos version 26 to analyze the relationships between variables to examine for correlation and construct mediated effects models.

**Results:**

Coping styles showed a significant positive correlation with family resilience (*r* = 0.347, *P* < 0.01) and a negative correlation with caregiver burden (*r* = -0.379, *P* < 0.01). A significant negative correlation was found between family resilience and caregiver burden (*r* = -0.503, *P* < 0.01). In the mediation model, patient coping styles directly impacted caregiver burden significantly (95% CI [-0.372, -0.058]), and coping styles indirectly impacted caregiver burden by family resilience in a significant way (95% CI [-0.275, -0.098]).

**Conclusions:**

Patient coping styles directly affect caregiver burden. Family resilience is a mediating variable between patients’ coping styles and the burden on caregivers.

## Background

Chronic kidney disease (CKD) affects an estimated 11–13.4% of the global population [1]. The progression of CKD to end-stage renal disease (ESRD) leads to retention of uremic toxins in the body, dyshomeostasis of the internal environment, and multiple organ damage [2]. Many patients with ESRD choose maintenance hemodialysis (MHD) as a life-saving measure [2–3]. The need for continuous hemodialysis can aggravate the stress caused by this chronic and irreversible disease on the patients and their caregivers [4–6]. The term caregiver refers to a member of the patient’s family who is involved in the patient’s care and helps them adapt to and manage their chronic illness [7, 8], without receiving any remuneration for their care [5]. Patients receiving MHD heavily depend on their caregivers for assistance in all aspects of their daily lives [9], including fulfilling dietary requirements, managing medications, scheduling dialysis appointments, and providing transportation to the hospital [1, 4, 10]. The limited self-care capabilities of these patients and the frequent complications of hemodialysis increase their reliance on caregivers, aggravating the stress on the caregivers [5, 11–14]. The term caregiver burden (CB) primarily refers to the adverse impact on a caregiver’s physical health or financial situation as a result of caring for a sick relative [1]. This burden stems from the prolonged and demanding caregiving responsibilities, which place significant financial, social, and psychological strain on the caregiver [1, 4, 14]. Understanding the determinants of the burden of care can help inform targeted measures to reduce the burden on caregivers [15].

Coping has been recognized as a vital variable in understanding an individual’s response to changing health and illness conditions [16]. Coping is a cognitive or behavioral process intended to manage or reduce an event that an individual perceives as affecting his or her well-being [15]. Lazarus and Folkman [17] categorized coping styles into two dimensions: positive coping and negative coping [18]. Positive coping strategies are manifested by actively seeking support from society or the resources of those around them, which helps individuals cope with stress more easily. Positive coping enables patients to achieve healthier psychological outcomes in the long term [19–21]. In contrast, negative coping strategies, such as avoidance or dissociation, can adversely affect an individual’s health and subjective well-being [18]. Negative coping only can temporarily alleviate stress by removing the caregiver from the stressful situation. However, avoidance is not the solution to the underlying cause. The adverse psychological coping and behaviors exhibited by patients can significantly impact the outcomes of disease treatment, leading to heightened patient dependence and exacerbating the caregiver’s responsibilities [12].

Since the inception of the Patient and Family Centered Care (PFCC) model, health systems have viewed family members as an integral part of patient care, incorporating family preferences, needs, and values into patient treatment [22]. A study suggested that close relative caregivers experience a heavier burden as family members than other non-relative caregivers. This may be attributable to the greater emotional involvement of family members in the caregiving process, which may make them feel obligated to care for family members even if their personal well-being is compromised [23]. Family resilience reflects the ability of family systems to cushion stress, recover from crises, reduce dysfunction and adapt to new circumstances [24].Resilience is a protective element of psychological health and an important pillar when coping with stress [24, 25]. Several biological, psychological, social, and cultural factors can influence resilience [26]. A study reported an association between patients’ coping styles and family support [27]. However, the relationship between family resilience, patient coping styles, and caregiver burden is not well characterized in contemporary literature.

The accumulation of caregiver burden can affect the quality of care for the patient and the treatment process, which can in turn aggravate the caregiver’s burden triggering a vicious cycle [5]. According to the stress process model [28, 29], the patient is the stress for the caregiver, family resilience can be considered a buffer in the stress process, and the caregiver burden is the stress response [30]. Therefore, in this study, we defined patient coping styles as the antecedent variable, family resilience as the mediator, and caregiver burden as the outcome variable. The research hypotheses were as follows: (H1) The coping strategies of patients directly influence the burden of caregivers. (H2) Family resilience plays a mediating role between the coping strategies of patients and the burden of caregivers.

## Methods

### Study design

This was a cross-sectional and descriptive-analytic study. A convenience sample of MHD patients and their primary caregivers was recruited from September 2022 to October 2022 at the Hemodialysis Center of Foshan First People’s Hospital in China.

### Study population

Inclusion criteria: (1) adults patients (age ≥ 18 years) who had been on regular dialysis for more than 3 months and had adult caregivers with at least a 3-month history of care for hemodialysis patients; (2) caregivers were immediate family members and were currently providing uncompensated care and support to the patients; (3) ability to read or express their views; (4) provision of voluntary consent for participation in the study.

Exclusion criteria:(1) patients with mental disorders diagnosed by a physician according to the Diagnostic and Statistical Manual of Mental Disorders (DSM-IV, TR) [31]; (2) inability to communicate orally; and (3) presence of other life-threatening comorbidities, such as malignant tumors, cardiorespiratory failure, and severe infection.

### Sample size

Referring to the sample rough estimation method proposed by Kendall et al [32], the scale entry with the highest number of entries was selected as the criterion of calculation, with a sample size of 5 to 10 times the entries of the scale, plus a 10% questionnaire invalidity rate. In this study, the Zarit Caregiver Burden Scale with the highest number of entries (22 entries) was used for calculation, resulting in a survey sample size of 121 to 242.

### Data collection

Patients and their caregivers filled out the paper-and-pencil self-reported questionnaires in two separate rooms with a quiet environment before the initiation of hemodialysis. All self-report assessments were conducted by well-trained, independent evaluators. The evaluators were all nurses from the hemodialysis unit, and all evaluators received uniform training prior to the investigation. Uniform instruction content was used to explain the method and precautions for questionnaire completion to the study participants. Patients capable of self-completion completed the questionnaire unassisted. For patients who had difficulty in writing, the interviewer assisted them in completing the questionnaire. These investigators simply read the items verbatim without any additional explanation. The time required to complete the survey was 15–20 min. The assessor reviewed the questionnaire immediately after its completion and asked participants to complete any missing items. At the end of the survey, a small gift was given to all participants as compensation for the time spent on completing the survey.

### Ethical considerations

The study was carried out at the Hemodialysis Center according to the revised Helsinki Declaration 2013, which was approved by the hospital’s Ethics Committee (No. 2,022,082). The purpose and procedures of the study were explained to all participants, and they were informed of their right to withdraw from the study at any time and refuse to answer any questions. Before the study began, all participants signed a written informed consent document, showing full knowledge of the study process.

### Survey tools

The survey consisted of four parts: (1) Sociodemographic characteristics of patients and caregivers, (2) Chinese version of the Simple Coping Style Questionnaire (SCSQ), (3) Chinese version of the 22-item version of the Zarit Burden Interview (ZBI-22), and (4) the Family Resilience Assessment Scale of Chinese version (C-FRAS). The SCSQ was completed by the patient; the ZBI-22 and C-FRAS were completed by the primary caregiver.

### Coping style

Folkman and Lazarus [35] developed the Coping Styles Scale to assess individuals’ coping styles. This graduate student used the Chinese version of the Simple Coping Style Questionnaire (SCSQ) [36], which has good reliabilities and validities in the population of China. It has 20 items, including 2 subscales: positive coping (12 items) and negative coping (8 items). The scale is based on a 4-point Liker scale ranging from not taking (0) to often taking (3). The higher the score is on a particular dimension; the more likely participants tend to adopt that particular coping style. The Cronbach’s alpha coefficient was 0.807 for this total scale and 0.730–0.847 for the subscales.

### Caregiver burden (ZBI)

Zarit and colleagues [37] developed the Caregiver Burden to assess caregivers’ burden. The Chinees version of Zarit Burden Interview (ZBI-22) [38], which has been tested in Chinese samples with good reliability and validity, was used in the current study. It consists of 2 sub-scales: individual burden (12 items) and liability burden (6 items), and the remaining 4 are independent scoring items, totaling 22 items. Each entry was scored on a 5-point Likert scale from none (0) to always (4), and the scale scores ranged from 0 to 88, with higher scores indicating greater caregiving burden. Each item was scored on a 0 to 4 scale from 0 to 88, with a total score of < 20 as no or very light burden, 20 to 39 as mild burden, 40 to 59 as moderate burden, and ≥ 60 as severe burden. The Cronbach’s alpha coefficient for the ZBI was 0.934, and the Cronbach’s alpha coefficients for the subscales ranged from 0.853 to 0.888.

### Family resilience

Family resilience was assessed using the Chinese version of the Family Resilience Assessment Scale (C-FRAS) [32], which has been examined in Chinese families and has good reliability and validity. It includes four subscales: perseverance (6 items), amicability (6 items), openness (4 items), and supportiveness (4 items), with a total of 20 items. The scale is based on a 5-point Likert scale ranging from very noncompliant (1 point) to very compliant (5 points) and the scale is between 20 and 100, with a higher score indicating more resilience in the family. The Cronbach alpha was 0.944 on the overall scale and 0.787 to 0.900 on the subscale.

### Sociodemographic information

Sociodemographic information included the patient’s age, gender, education level, marital status, residency status, work status and type of health insurance. For the primary caregiver, data regarding age, gender, marital status, education level, work status, duration of caregiving, self-perceived health status, chronic illnesses and monthly household income were collected.

### Statistical methods

The study was statistically analyzed using IBM SPSS 23.0 and Amos 26.0. Descriptive statistics (means and frequency percentages) and analytical statistics (Pearson correlation and model construction) were used. Because our data were nearly normally distributed, independent samples t-tests or one-way analysis of variance (ANOVA) were used to compare the score of caregiver’s CB by demographic features. Pearson correlation analysis was used to explore the correlation between family resilience, caregiver burden, and patient coping styles. Amos 26.0 was used for mediation effect analysis and model construction, and the significance of the mediation model was examined using the bias-corrected percentile bootstrap method (replicate sampling of 5000, 95% CI), with the level of statistical significance set at less than 0.05 (two-tailed).

## Results

### Tests of normality

In Table 1, Kolmogorov–Smirnova and Shapiro–Wilk test indicate a significance level (Sig.) of greater than 0.05, suggesting conformity with normal distribution standards for caregiver burden scores.

**Table 1:** Tests of Normality

	Kolmogorov-Smirnova			Shapiro-Wilk		
	Statistic	Degree of freedom(df)	Sig.	Statistic	df	Sig.
Caregiver Burden Scores	0.056	173	0.200	0.990	173	0.244

### Distribution of individual characteristics and differences in caregiver burden and household resilience

One hundred and seventy-three pairs of patients and their caregivers participated of this study. Table 2 shows the individual attributes of patients, and the relationship between the patients’ individual attributes and caregiver burden. In the 173 patients,65 (37.6%) patients were aged 45–64 years, and 64 (37.0%) patients were aged 65–79 years. 95 (54.9%) patients were male, and 78 (45.1%) patients were female. Regarding marital status, 79.2% of the patients were married. In terms of educational level, 98 (54.9%) patients had an educational level of primary school or below.79.2% were retired or unemployed, and 93.1% lived with their families.

Table 3 shows the individual attributes of caregivers, and the relationship between the caregivers’ individual attributes and caregiver burden. Most of the caregivers were above 45 years old (*n* = 117, 67.6%), 76 (43.9%) caregivers were aged 45–64 years old, 37 (21.4%) caregivers were aged 65–79 years old, another 50.3% caregivers were females, and 82.1% caregivers were married. Regarding education level and working status, 68 (39.3%) caregivers had primary school or below, and 74 (42.8%) caregivers were in full-time employment. 92(53.2%) caregivers had been caring for hemodialysis patients for more than 5 years. Besides, 38(22%) caregivers had a chronic illness, and 104(60%) caregivers were spouses of the patients.

Analysis of the relationship between individual characteristics and burden showed that caregiver burden was statistically significantly different in terms of caregiver marital status, monthly household income, self-perceived health status, and type of patient health insurance.

**Table 2:** Basic data and one-way ANOVA of MHD patients(*n* = 173 )

Variables	patients	caregiver burden	
	N(%)	Mean(SD)	t/F
Age			
19-44Y	33(19.1)	23.6(16.0)	0.604
45-64Y	65(37.6)	24.6(16.3)	
65-79Y	64(37.0)	21.7(14.6)	
≥ 80Y	11(6.3)	27.3(19.8)	
Gender			
Male	95(54.9)	25.59(16.0)	3.273
Female	78(45.1)	21.4(15.4)	
Education			
Primary school or below	95(54.9)	25.4(16.2)	2.173
Secondary school	54(31.2)	19.8(13.9)	
University or above	24(13.9)	24.5(17.5)	
Marital status			
Single	9(5.2)	20.2(13.8)	0.939
Married	137(79.2)	24(16.0)	
Widowed/divorced	27(15.6)	22(15.5)	
Work status			
Employed	29(16.8)	20.2(16.5)	1.143
Part time	7(4.0)	24.6(15.5)	
Retired	87(50.3)	22.8(15.7)	
Unemployed	50(28.9)	26.6(15.5)	
Residency status			
with family	161(93.1)	23.3(15.9)	0.651
Alone	12(6.9)	27.1(15.4)	
Type of health insurance			
Employee/resident health insurance/social insurance	146(84.4)	23.1(15.7)	3.755*
New Rural Cooperative Medical Care	21(12.1)	21.8(14.9)	
Self-financed	6(3.5)	40.5(15.3)	

*p<0.05; **p<0.01

**Table 3:** Basic data and one-way ANOVA of caregivers(*n* = 173)

Variables	caregivers	caregiver burden	
	N(%)	Mean(SD)	t/F
Age			
19-44Y	56(32.4)	20.8(16.3)	1.23
45-64Y	76(43.9)	24.6(1.8)	
65-79Y	37(21.4)	26.1(15.4)	
≥ 80Y	4(2.3)	16.8(15.1)	
Gender			
Male	86(49.7)	23.3(16.3)	0.031
Female	87(50.3)	23.7(15.4)	
Education			
Primary school or below	68(39.3)	25.1(15.1)	0.676
Secondary school	55(31.8)	23.3(16.8)	
University or above	50(28.9)	21.7(15.7)	
Marital status			
Single	19(11.0)	18.4(13.8)	4.436*
Married	142(82.1)	23.2(15.1)	
Widowed/divorced	12(6.9)	35.2(21.8)	
Work status			
Employed	74(42.8)	19.9(14.4)	2.484
Part time	17(9.8)	26.8(20.2)	
Retired	57(32.9)	26.9(16.0)	
Unemployed	25(14.5)	24.4(14.8)	
Monthly household income per capita (RMB)			
<3000	33(19.1)	24.6(15.6)	3.161*
3000 ~ 6000	85(49.1)	25.6(15.4)	
6001 ~ 10,000	34(19.7)	23.1(17.5)	
>10,000	21(12.1)	14.1(12.0)	
Self-perceived health status			
Bad	4(2.3)	34.0(11.9)	5.939**
General	88(50.9)	26.9(15.8)	
Well	81(46.8)	19.4(15.0)	
Suffer from at least one chronic disease (hypertension, diabetes, etc.)			
Yes	38(22.0)	25.9(16.1)	0.105
No	135(88.0)	22.9(15.7)	
Duration of caregiving			
3 months<1 years	27(15.6)	23.3(16.6)	0.492
1 ~ 3 years	31(17.9)	21.6(16.1)	
3 ~ 5 years	23(13.3)	21.3(17.7)	
>5 years	92(53.2)	24.8(15.1)	

*p<0.05; **p<0.01

### Distribution of family resilience, coping styles, and caregiver burden scores

The results of the ZBI scale showed that the burden score for caregivers was 23.53 ± 15.81, indicating a mild caregiving burden for hemodialysis caregivers. The family resilience score was 77.49 ± 11.55, indicating a moderate level of family resilience in hemodialysis patients. The difference between the positive and negative coping dimensions was 0.29 ± 0.75 and scores higher than 0 indicated a preference for positive coping styles, which showed that hemodialysis patients were more inclined to adopt positive responses.

### Correlations among family resilience, coping styles, and caregiver burden

Table 4 shows significant correlations of family resilience, coping styles and caregiver burden among MHD patients. The results indicated that there was a positive correlation between coping styles and family resilience (*r* = 0.347, *P* < 0.01). There was a negative correlation between the burden of caregivers and family resilience (*r* = -0.503, *P* < 0.01). There was a negative correlation between the burden of caregiver and the coping styles (*r* = -0.379, *P* < 0.01). This finding suggests that the more patients’ coping styles tend to adopt positive coping styles, the lower their caregiver burden.

**Table 4:** Correlation among coping styles, family resilience, and caregiver burden

Variable	Coping styles	Family resilience	Supportiveness	Openness	Amicability	Toughness	Burden	Responsibility burden	Personal burden
Coping styles	1.000								
Family resilience	0.347^**^	1.000							
Supportiveness	0.242^**^	0.787^**^	1.000						
Openness	0.323^**^	0.821^**^	0.603^**^	1.000					
Amicability	0.307^**^	0.893^**^	0.630^**^	0.620^**^	1.000				
Toughness	0.305^**^	0.902^**^	0.595^**^	0.648^**^	0.738^**^	1.000			
Burden	-0.379^**^	-0.503^**^	-0.342^**^	-0.479	-0.447	-0.442	1.000		
Responsibility burden	-0.350^**^	-0.511^**^	-0.322^**^	-0.482^**^	-0.476^**^	-0.446^**^	0.922	1.000	
Personal burden	-0.374^**^	-0.432^**^	-0.304^**^	-0.407^**^	-0.392^**^	-0.367^**^	0.963	0.810^**^	1.000

**p<0.01

### Mediation model construction

**Fig. 1:**
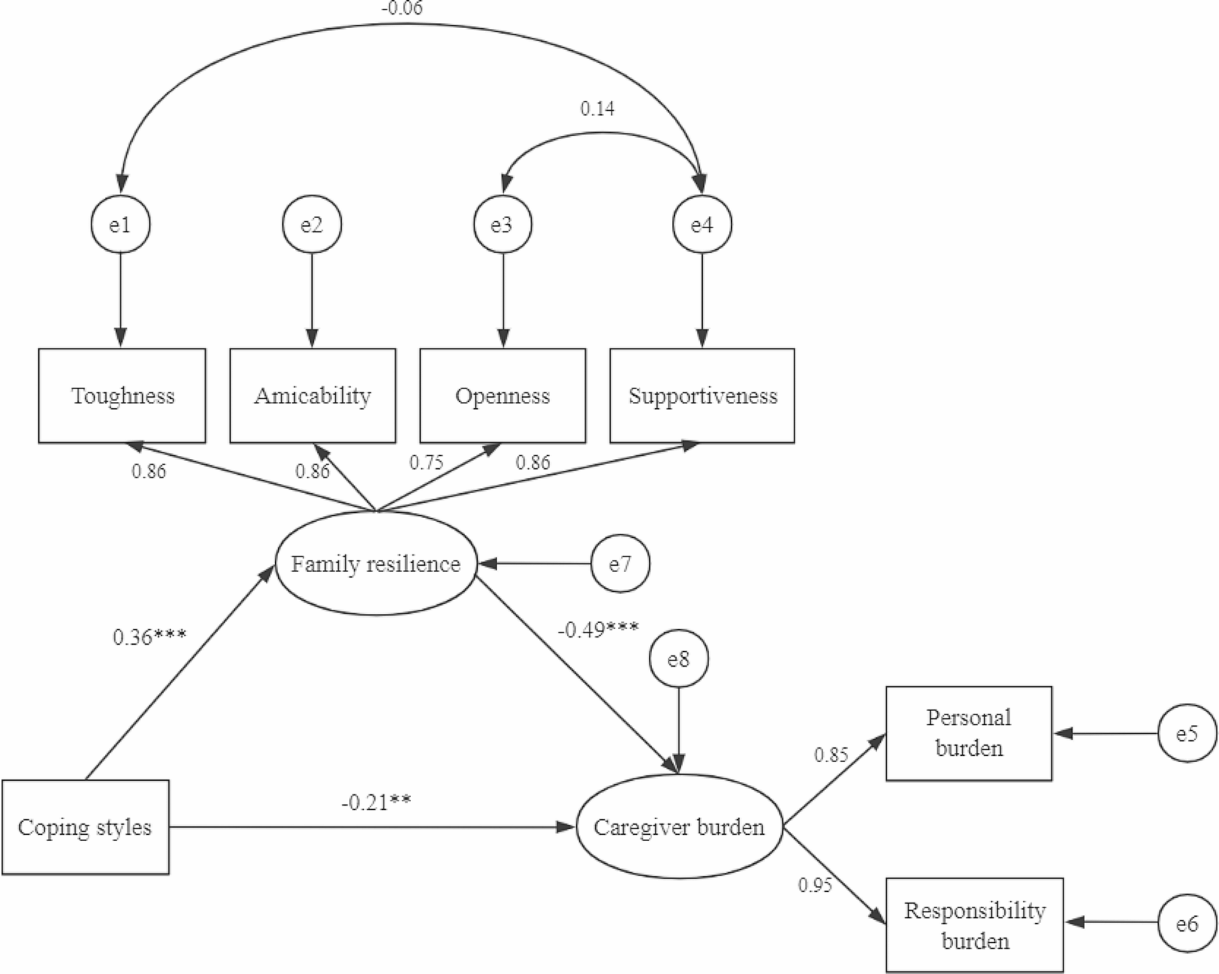
The SEM of predictors of coping styles, family resilience and caregiver burden

From the analysis of the three correlations, there is a significant correlation between coping styles, family resilience and caregiver burden, so mediation effect analysis can be performed. According to the validation procedure for mediating effects, to investigate the direct and indirect effects of patients’ coping styles and family resilience on caregiver burden this study chose the AMOS 26.0 software to build a model (Fig. 1). The latent variable family resilience was estimated with 4 dimensions of perseverance, amicability, supportiveness, and openness as exogenous variables. The latent variable burden was estimated with 2 dimensions of personal burden and burden of responsibility as exogenous variables. Coping styles was then directly used as an exogenous variable. The results are shown in Fig. 1. Overall quality of fit statistics demonstrated that the proposed model was a good match for CMIN/DF (Chi-square Degrees of Freedom Ratio) of 1.353, a RMSEA (Root Mean Square of Error) of 0.045, a GFI (Goodness of Fit Index) of 0.979, an AGFI (Adjusted Goodness of Fit Index) of 0.942, and a CFI (Comparative Fit Index) of 0.994. Figure 1 shows that family resilience is the mediating variable between patients’ coping styles and caregiver burden.

### Path testing for structural equation model

In the path analysis of structural equation modeling, coping style could positively predict family resilience (β = 0.365, *p* < 0.001) and could negatively predict caregiver burden (β=-0.208, *p* = 0.005). In contrast, family resilience could negatively predict caregiver burden (β=-0.485, *p* < 0.001). Table 5; Fig. 1 present the results.

### Mediating effects of family resilience

Indirect effects among coping style, family resilience and caregiver burden were analyzed by performing a bootstrap with 5000 resamples and a bias-corrected and Percentile confidence interval of 95%. Table 6 indicated that patient coping styles directly influenced caregiver burden, with a direct effect size of -0.208 (95% CI, -0.372 to -0.058) and family resilience was a significant mediating variable for patient coping styles and caregiver burden, with an indirect effect size of -0.177 (95% CI, -0.275 to -0.098). The effect percentage calculations in Table 6 show that family resilience, as a mediating factor, had an indirect effect size of 45.97%, and the direct effect size of patient coping styles was 54.03%. Therefore, family resilience has an indirect impact on the relationship between the coping styles and the burden of care.

**Table 5:** Standard and non-standard coefficients in the proposed path model

Path relationship test	Std	Unstd	S.E.	C.R.	P
Coping styles → Family resilience	0.365	1.776	0.378	4.698	<0.001
Family resilience → Caregiver Burden	-0.485	-0.944	0.17	-5.539	<0.001
Coping styles → Caregiver Burden	-0.208	-1.968	0.699	-2.815	0.005

Std = standardized regression coefficients, Unstd = unstandardized regression coefficient

**Table 6:** Bootstrap mediated effects results

Parameter	Estimate	95% CI		P	efficiency ratio (%)
		Lower	Upper		
Indirect effects	-0.177	-0.275	-0.098	<0.01	45.97
Direct effects	-0.208	-0.372	-0.058	<0.01	54.03
Total effects	-0.385	-0.518	-0.243	<0.01	100

***P < 0.001, **P < 0.01

## Discussion

The mean caregiver burden score in this study was 23.53 ± 15.81, indicating a mild caregiving burden. This result is consistent with the results reported by Alshammari et al. [33] and Dongju Yang et al. [34]. However, it was lower than that reported by Mona et al. [9], which may be related to differences with respect to the study population, inclusion criteria, and sample size. The burden of care on caregivers can be influenced by many factors. The study revealed significant differences in caregiver burden based on marital status, monthly income, self-perceived health status, and type of patient health insurance. Diao et al [35] observed significant differences in ZBI-22 scores between primary caregivers with different income levels. Jafari et al [5] also reported that caregivers of low-income patients were experiencing higher levels of care burden. This may be because long-term MHD patients consume more family wealth as a result of the treatment of the disease. Additionally, the result indicated that There were significant differences in ZBI-22 scores among caregivers with different self-reported health conditions. Caregivers’ health conditions, coupled with the pressure of caring for hemodialysis patients, can aggravate the burden of care. This finding is consistent with Niko et al. reporting that higher levels of burden are associated with poorer physical health [36]. Therefore, more attention and support need to be given to caregivers with health problems [37]. Finally, the study showed that the caregiver burden score was significantly higher for widowed/divorced people. Widowed/divorced people were less likely to receive material and emotional support from their spouses than those who were married. Conversely, marital unions can provide higher levels of social support. Thus, it is essential that widowed or divorced caregivers receive targeted emotional or psychosocial support to improve their mental health.

Our study investigated the impact of patient coping styles on caregiver burden and explored the mediating role of family resilience from the perspectives of the patient, caregiver, and family. Our findings indicate that the patient’s coping style directly influence caregiver burden. A previous study has emphasized the significance of patients’ coping mechanisms as primary stressors and predictors of caregiver burden, underlining how patients’ attitudes toward their condition can shape the psychological and social challenges encountered by caregivers [38]. A positive coping approach embraced by patients can significantly alleviate their caregivers’ stress, serving as a motivational factor in stress management [39]. Conversely, negative coping by patients amplifies the demands of caregiving, aggravating the burdens associated with daily life and social interactions, thereby elevating the risk of mental health issues such as depression and anxiety [1]. This, in turn, compromises the quality of care provided [5]. These findings are consistent with those reported by Alshammari et al., who found that the patient’s attitude toward their illness, the duration of care, and the nature of the patient’s treatment impact the caregiver’s burden [33, 37]. Therefore, healthcare professionals can alleviate caregiver burden by evaluating and addressing patients’ coping styles through cognitive-behavioral therapy. Increased patient education and provision of psychological support can promote constructive coping strategies in dealing with the disease.

Our study found that family resilience has direct and indirect effects on reducing the burden on the caregivers of hemodialysis patients. The family, as a shared environment, acts as a crucial link between the patient and caregiver, aligning with the patient- and family-centered care model. Studies have shown that sharing caregiving tasks with family members reduces burden, emphasizing the role of family support in alleviating both physical and psychological burdens on the caregiver [6, 13, 40]. Favorable family relations and a pleasant home atmosphere can help reduce stress and promote the well-being of caregivers. Maintaining family harmony and strengthening family resilience improves caregiver-patient relationships and reduces their psychological and life burdens [6]. Additionally, caregivers with weaker family relationships and a colder family environment have fewer coping resources, aggravating the burden. The intermediary model shows that family resilience mediates the relationship between patient coping styles and caregiver burden, echoing the findings of Yuli Li et al. [41]. These findings suggest that family resilience moderates the link between a patient’s coping style and the caregiver’s perceived burden. The Caregiver Stress Theory (CST) [40] based on Roy’s Adaptation Theory explains the psychological responses of caregivers to caregiving tasks. According to this theory, caregiver burden is related to caregiving task-related products, such as caregiving arrangements and timing, social roles, and family support [6, 42]. Therefore, encouraging other family members to share the patient’s concerns and participate in caregiving can help augment the family’s ability to combat difficulties together and improve the quality of life of the patient and caregiver.

### Limitations

Some limitations of this study should be acknowledged. First, our study demonstrates the mediating role of family resilience in the relationship between patient coping styles and caregiver burden; however, due to the cross-sectional design, this study was unable to determine the long-term impact of patients’ coping styles on caregivers. A future study with longitudinal data collection can help overcome this limitation. Second, the caregivers in this study were relatives of the patients. Therefore, caregivers were generally reluctant to leave caregiving tasks to others or to complain about their caregiving roles. Caregiver questions have been biased by subjectivity. In addition, owing to the small sample size, larger studies are required to obtain more robust results. Finally, this study assessed family resilience based only on caregiver self-reports, which may differ from the level of resilience of the entire family. Future research should explore family resilience from the perspective of all family members.

### Conclusion

Patient coping styles directly affect caregiver burden. Family resilience indirectly influences the effect of patient coping styles on caregiver burden. In this study, patients’ negative coping was found to have a negative effect on caregiver burden and family resilience showed a positive effect on caregiver burden. Our findings suggest that healthcare professionals should assess the patients’ coping styles and implement interventions to mitigate the adverse effects of negative coping on the caregiver burden. The positive impact of family resilience should be fully leveraged to regulate the relationship between patients and caregivers in order to reduce the caregiver burden associated with patients’ negative coping styles.

## Data Availability

The original data of this study will be provided by the authors, and please contact the corresponding author if any further questions.

## Abbreviations

CKD: Chronic kidney disease
ESRD: End-stage renal disease
MHD: Maintenance hemodialysis
CSSQ: Chinese version of the Simple Coping Style Questionnaire
CB: Caregiver burden
ZBI-22: The 22-item version of the Zarit Burden Interview
C-FRAS: the Family Resilience Assessment Scale of Chinese version (C-FRAS)

## References

[1] Adejumo OA, Iyawe IO, Akinbodewa AA, Abolarin OS, Alli EO (2019). Burden, psychological well-being and quality of life of caregivers of end stage renal disease patients. Ghana Med J.

[2] Power A, Duncan N, Goodlad C (2009). Advances and innovations in dialysis in the 21st century. Postgrad Med J.

[3] Lahtinen J, Alhava EM, Aukee S (1978). Acute cholecystitis treated by early and delayed surgery. A controlled clinical trial. Scand J Gastroenterol.

[4] Hoang VL, Green T, Bonner A (2019). Informal caregivers of people undergoing haemodialysis: associations between activities and burden. J Ren care.

[5] Jafari H, Ebrahimi A, Aghaei A, Khatony A (2018). The relationship between care burden and quality of life in caregivers of hemodialysis patients. BMC Nephrol.

[6] Alshammari B, Noble H, McAneney H, Alshammari F, O’Halloran P. Caregiver Burden in Informal Caregivers of Patients in Saudi Arabia Receiving Hemodialysis: A Mixed-Methods Study. Healthcare (Basel, Switzerland). 2019;11(3). 10.3390/healthcare11030366 PMC9914672 36766941

[7] Gilbertson EL, Krishnasamy R, Foote C, Kennard AL, Jardine MJ, Gray NA (2019). Burden of Care and Quality of Life among caregivers for adults receiving maintenance Dialysis: a systematic review. Am J Kidney Diseases: Official J Natl Kidney Foundation.

[8] Qiu Y, Huang Y, Wang Y, Ren L, Jiang H, Zhang L (2021). The role of Socioeconomic Status, Family Resilience, and Social Support in Predicting Psychological Resilience among Chinese maintenance hemodialysis patients. Front Psychiatry.

[9] Abed MA, Khalifeh AH, Khalil AA, Darawad MW, Moser DK (2020). Functional health literacy and caregiving burden among family caregivers of patients with end-stage renal disease. Res Nurs Health.

[10] Suri RS, Larive B, Hall Y, Kimmel PL, Kliger AS, Levin N (2014). Effects of frequent hemodialysis on perceived caregiver burden in the frequent Hemodialysis Network trials. Clin J Am Soc Nephrology: CJASN.

[11] Kuang Y, Wang M, Yu NX, Jia S, Guan T, Zhang X (2023). Family resilience of patients requiring long-term care: a meta-synthesis of qualitative studies. J Clin Nurs.

[12] Nagarathnam M, Sivakumar V, Latheef SAA (2019). Burden, coping mechanisms, and quality of life among caregivers of hemodialysis and peritoneal dialysis undergoing and renal transplant patients. Indian J Psychiatry.

[13] Alshammari B, Noble H, McAneney H, Alshammari F, O’Halloran P. Factors Associated with Burden in caregivers of patients with end-stage kidney disease (a systematic review). Healthc (Basel Switzerland). 2021;9(9). 10.3390/healthcare9091212 PMC8468425 34574986

[14] Joseph SJ, Bhandari SS, Dutta S, Khatri D, Upadhyay A (2021). Assessing burden and its determinants in caregivers of chronic kidney disease patients undergoing haemodialysis. Open J Psychiatry Allied Sci.

[15] Halcomb E, Fernandez R, Mursa R, Stephen C, Calma K, Ashley C (2022). Evaluation of the brief coping orientation to problems experienced scale and exploration of coping among primary health care nurses during COVID-19. J Nurs Adm Manag.

[16] Roy C, Bakan G, Li Z, Nguyen TH (2016). Coping measurement: creating short form of coping and Adaptation Processing Scale using item response theory and patients dealing with chronic and acute health conditions. Appl Nurs Research: ANR.

[17] Lazarus RS (1993). Coping theory and research: past, present, and future. Psychosom Med.

[18] Otto J, Linden M (2020). Stress coping styles among patients in a psychosomatic setting. Fortschr Neurol Psychiatr.

[19] Alkrisat M, Dee V (2014). The validation of the Coping and Adaptation Processing Scale based on the Roy adaptation model. J Nurs Meas.

[20] Carver CS, Pozo C, Harris SD, Noriega V, Scheier MF, Robinson DS (1993). How coping mediates the effect of optimism on distress: a study of women with early stage breast cancer. J Personal Soc Psychol.

[21] Kannis-Dymand L, Hughes E, Mulgrew K, Carter JD, Love S (2020). Examining the roles of metacognitive beliefs and maladaptive aspects of perfectionism in depression and anxiety. Behav Cogn Psychother.

[22] Kumpf VJ, Neumann ML, Kakani SR (2023). Advocating for a patient- and family centered care approach to management of short bowel syndrome. Nutr Clin Practice: Official Publication Am Soc Parenter Enter Nutr.

[23] Saltzman WR, Pynoos RS, Lester P, Layne CM, Beardslee WR (2013). Enhancing family resilience through family narrative co-construction. Clin Child Fam Psychol Rev.

[24] Lenz A (2016). [Family resilience and family therapy]. Praxis Der Kinderpsychologie Und Kinderpsychiatrie.

[25] Llistosella M, Castellvi P, Miranda-Mendizabal A, Recoder S, Calbo E, Casajuana-Closas M et al. Low resilience was a risk factor of Mental Health problems during the COVID-19 pandemic but not in individuals exposed to COVID-19: a Cohort Study in Spanish Adult General Population. Int J Environ Res Public Health. 2022;19(22). 10.3390/ijerph192215398 PMC9690878 36430116

[26] Southwick SM, Bonanno GA, Masten AS, Panter-Brick C, Yehuda R. Resilience definitions, theory, and challenges: interdisciplinary perspectives. Eur J Psychotraumatology. 2014;5. 10.3402/ejpt.v5.25338 PMC4185134 25317257

[27] Diong SM, Bishop GD, Anger Expression (1999). Coping styles, and well-being. J Health Psychol.

[28] Thoits (1995). Stress, coping, and social support processes: where are we? What next?. J Health Soc Behav.

[29] Pearlin LI (1989). The sociological study of stress. J Health Soc Behav.

[30] Alexander T, Wilz G (2010). Family caregivers: gender differences in adjustment to stroke survivors’ mental changes. Rehabil Psychol.

[31] Association AP. Diagnostic and Statistical Manual of Mental Disorders DSM-IV-TR Fourth Edition (Text Revision). 2001.

[32] Kendall PC, Sheldrick RC (2000). Normative data for normative comparisons. J Consult Clin Psychol.

[33] Alshammari B, Noble H, McAneney H, Alshammari F, O’Halloran P. Caregiver Burden in Informal caregivers of patients in Saudi Arabia receiving hemodialysis: a mixed-methods study. Healthc (Basel Switzerland). 2023;11(3). 10.3390/healthcare11030366 PMC9914672 36766941

[34] Li D, Jiang X (2017). Studies on the care burden of primary caregivers for maintenance hemodialysis patients. Chin J Blood Purif.

[35] Diao H, Ji Y, Chen R, Zhang T, Li X. Analysis of the burden of main caregivers and its influencing factors in middle aged and elderly patients receiving maintenance hemodialysis. Med High Vocat Educ Mod Nurs. 2020(04):313–6.

[36] Kristianingrum ND, Ramadhani DA, Hayati YS, Setyoadi S. Correlation between the burden of family caregivers and health status of people with diabetes mellitus. J Public Health Res. 2021;10(2). 10.4081/jphr.2021.2227 PMC8129740 33855412

[37] Vovlianou S, Koutlas V, Ikonomou M, Vassilikopoulos T, Papoulidou F, Dounousi E (2023). Quality of life of caregivers of end-stage kidney disease patients: caregivers or care recipients?. J Ren care.

[38] Tsai P-F. Development of a middle-range theory of caregiver stress from the Roy Adaptation Model. Wayne State University, Detroit, Michigan; 1998.

[39] Menati L, Torabi Y, Andayeshgar B, Khatony A (2020). The relationship between Care Burden and coping strategies in caregivers of Hemodialysis patients in Kermanshah, Iran. Psychol Res Behav Manage.

[40] Tsai PF (2003). A middle-range theory of caregiver stress. Nurs Sci Q.

[41] Li Y, Qiao Y, Luan X, Li S, Wang K (2019). Family resilience and psychological well-being among Chinese breast cancer survivors and their caregivers. Eur J Cancer Care.

[42] Zhang W, Gao Y, Ye M, Zhou W, Zhou L (2023). Family resilience and its predictors among patients with a first-ever stroke one month after stroke: a cross-sectional study. Top Stroke Rehabil.

